# Structural violence in South African primary healthcare facilities: insights from discussions with adolescents and young people seeking sexual and reproductive health needs

**DOI:** 10.1080/17482631.2022.2056955

**Published:** 2022-03-27

**Authors:** Mokhantšo Makoae, Tsidiso Tolla, Zitha Mokomane, Tholang Mokhele

**Affiliations:** a Developmental Capable and Ethical State, Human Sciences Research Council, Pretoria, South Africa; b Public Health and Family Medicine, University of Cape Town, Rondebosch, South Africa; c Sociology Department, University of Pretoria, Pretoria, South Africa

**Keywords:** Structural violence, adolescents and youth, sexual and reproductive health, primary healthcare, South Africa

## Abstract

Introduction. South Africa has an enabling legislative and policy framework that promotes the protection of adolescents and young people’s sexual and reproductive health and rights. Much of the literature in this field has identified discriminatory and hostile attitudes from healthcare workers as a major underlying factor to negative sexual and reproductive health outcomes for this age cohort. Not as well understood is the role of structural violence although this type of violence, through its structures of injustice and inequalities, is closely associated with stigma and discrimination. Data and sources. To contribute to closing this research gap, this paper draws on the findings of a larger qualitative study, specifically focus group discussions with young people aged 15–24 years. Results. The consequences of these attitudes within the structural violence framework are illuminated as are recommendations for enhancing access to sexual and reproductive health and services by adolescents and young people. Discussion and conclusion. Key among the latter is that young people’s sexual and reproductive health needs and wellbeing should be pursued through a multisectoral approach that encompasses stigma reduction interventions involving the young people, families, and communities collaborating with healthcare workers.

## Introduction

South Africa has a comprehensive and enabling legislative and policy framework for the provision of sexual and reproductive health services to adolescents and young people (AYP). This framework is also widely seen as providing a prototype of adolescent and youth-friendly health services (AYFS) through various settings including schools, communities and healthcare facilities (MiET Africa [Internet], 2011; Tylee et al., 2007). Despite this, this age cohort—which comprises of adolescents in the age group between 10 and 19 years old as well youth in the 20–24 age group —often faces a number of challenges in accessing these services in South Africa. Among the most widely documented challenges are negative experiences due to perceived unsupportive attitudes and poor rapport of healthcare workers (Mulaudzi et al., 2018; Schriver et al., 2014). The consequences of this limited access are reflected in, for example, early age of sexual debut; high levels of teenage pregnancy, unwanted pregnancies, and unsafe abortions; low and inconsistent use of condoms; low contraceptive prevalence; high prevalence of sexually transmitted infections (STIs) including HIV; and school dropout (Beksinska et al., 2014; Dellar et al., 2015; Geary et al., 2014, 2015; Ndaba, 2021; Schriver et al., 2014; Shisana et al., 2014).

Among the consistently noted factors for AYP’s limited access to sexual and reproductive health services is social stigma. Goffman (1963, p. 13) provides the classical description of stigma as “an attribute that is significantly discrediting” of individuals and is perceived by society as undesirable difference or some form of deviance in behaviour. Despite the ubiquitous evidence on the existence of stigma around young people’s sexuality in Sub-Saharan Africa (Abubakari et al., 2020) current stigma reduction interventions in these public healthcare facilities in South Africa tend to focus on specific diseases or conditions, and not groups such as AYP who seek access to sexual and reproductive health services. This can create challenges for effective access to sexual and reproductive health services as social stigma can render adolescents and young people disempowered to bring their health concerns such as exposure to pregnancy, sexually transmitted infections (STIs) including HIV, and sexual abuse to the attention of healthcare workers (Abubakari et al., 2020; Nyblade et al., 2019). This is essentially because, for a number of socio-cultural reasons, young people view healthcare workers not only as authority figures but also as having more conservative views in relation to young people’s sexuality and use of sexual and reproductive health services (Mokomane et al., 2017; Suleiman et al., 2017).

It is noteworthy, however, that adolescents and young people are in a unique phase in the life course characterized by significant physical, psychosocial, and cognitive changes. It is in this phase that young people also learn about intimate relationships and acquire biosocial competencies required for sexual activity and/or reproduction (Chandra-Mouli et al., 2015; Suleiman et al., 2017). Understanding these changes provides novel opportunities for engaging young people as they develop and can greatly reduce the engagement in risky sexual behaviour that leads to poor health outcomes (Kleinert & Horton, 2016). The engagement with young people to minimize the risks of negative sexual and reproductive health outcomes requires various key role players including healthcare workers (Healthcare workers) to recognize the far-reaching consequences of limiting young people’s access to sexual and reproductive health services. Of particular importance is to understand how, from human rights and human security perspectives, discouraging AYP from accessing sexual and reproductive health services constitutes unethical conduct that places young people in the way of harm and diminishes their agency (United Nations, 2014). This type of conduct, which can also be described as structural violence, constitutes a missed opportunity for ensuring that South Africa’s national commitment to address sexual and reproductive health challenges of AYP is realized.

Coined in 1969 by Johan Galtung, the concept of structural violence refers to forms of violence experienced in social structures or institutions by people of less power and status (based on characteristics such as gender, race or age) when they are prevented from meeting their basic needs. Central to this conceptualization is a distinction between avoidable and unavoidable harm that is inflicted in the context of unequal power, resources and opportunities and that, as a result, leads to unequal life chances for marginalized groups (Galtung, 1969). The concept thus advances the idea that unequal social relations and arrangements that define how individuals and groups experience social systems in their daily interactions can be depicted by suffering which is brought by institutions when they normalize injustices against individuals and groups (Rylko-Bauer & Farmer, 2016). To this end, it can be argued that one of the mechanisms by which healthcare workers in South Africa inflict structural violence is social stigma towards AYP seeking sexual and reproductive health services. Stigma does not only preclude opportunities for provider-initiated services, but it also constrains the latter’s “capabilities and agency, assaults their dignity, and sustains inequalities” that sustain structural violation of human rights (Rylko-Bauer & Farmer, 2016, p. 2). As such, it should be considered a violent action, which perpetuates avoidable suffering and does not promote human development and rights. Indeed, as Rylko-Bauer and Farmer argue, structural violence redefines the notion of risk by illustrating that the structures that perpetuate it “are violent because they result in avoidable deaths, illness, and injury; and they reproduce violence by marginalizing people and communities, constraining their capabilities and agency, assaulting their dignity, and sustaining inequalities” (Rylko-Bauer & Farmer, 2016, p. 45).

Given its potential to further enhance the understanding of AYP’s limited access to sexual and reproductive health services, this paper draws on the structural violence framework to raise critical questions about the experiences AYP who seek sexual and reproductive health services from primary healthcare facilities in the South African public sector, the largest provider of such services to the country’s population. The main research question is: to what extent do attitudes of healthcare workers towards young people seeking sexual and reproductive health services in South Africa constitute structural violence? With this focus, we believe that the paper is a novel contribution to this field as it provides critical insights into the perceived meanings of common healthcare workers’ discriminatory attitudes, practices, and behaviours as well as their consequences for AYP seeking sexual and reproductive health services.

## Data sources

This paper used data from a larger qualitative study aimed at providing an in-depth analysis of the programming and implementation of adolescent and youth sexual and reproductive health services in South Africa, as well as to illuminate the factors facilitate and those that inhibit effective delivery of such services in the country. The study was conducted in 2014 in selected districts in the country’s nine provinces. There were two main categories of study participants. The first was key informants (healthcare workers at different levels) in public health facilities that offer basic primary healthcare services. These included as clinics, hospitals, mobile clinics, community health centres, and satellite clinics.

The second category, on which this paper specifically draws from, was AYP aged 15–24 years who had sought sexual and reproductive health services at public health facilities in the selected districts—at least twice in the previous 2 years. This criterion meant to ensure that the young people still had vivid recollections of their experiences and perspectives of using public health facilities to access sexual and reproductive health services.

Data from these young people was obtained using focus group discussions (FGDs), qualitative data collection method that entail gathering together people from similar backgrounds or experiences to discuss a specific topic or to express their views, opinions or beliefs towards a focused topic (Bender & Eubank, 1994; Mishra, 2016). The main objective of FGDs in the qualitative study was to provide a forum for adolescents and youth to provide accounts of their experiences and perceptions regarding AYFS programming and implementation in their districts as well as to explore recommendations for improvement of service delivery. Specific focus was on facilities that were purposively selected in each district to represent the pilot National Health Insurance (referred to as “NHI facilities”) scheme and those identified as “non-NHI” facilities; those offering specific sexual and reproductive health services for AYP and those not offering these services; and those in rural areas, urban areas, and informal settlements or peri-urban areas. Participants for the FDG were recruited using purposive sampling techniques, specifically the snowballing method, which also ensured that the groups were heterogeneous, constituting young people from different socio-economic strata of society. It is noteworthy that the AYP served by the selected facilities came, almost invariably, from the Black African population, and to a smaller extent the Coloured population. While this is certainly a limitation as the data could not be analysed by sub-population—as often done in South Africa to depict the legacy of apartheid—the data derived from the FGDs are sufficient for the purposes of this paper considering that in South Africa, Black Africans are more likely to use public health facilities than other race groups (Naidoo, 2012). All discussions were conducted in the vernacular of each district and were audio-recorded with the consent of all the participants.

All in all, a total of 16 FGDs, each having between eight and ten participants (*n* = 87), were conducted by trained young male and female researchers. Although the larger study covered all nine provinces, FGDs with young people were not conducted in one province (Mpumalanga), due to logistical and administrative challenges that hampered the recruiting of participants in the timeframe allocated for data collection phase of the study. Despite this, saturation was reached through data collected in eight provinces.

At the end of the data collection phase, all the FGDs were transcribed verbatim and translated into English. To ensure accurate translation and capturing, the transcripts when then back translated to the vernacular. Thereafter, MM and ZM analysed the data using the first six of Colaizzi’s seven steps method of data analysis (Colaizzi, 1978). As articulated in [Authors, 2017], this entailed the following six steps, in accordance with Goulding (2005):

1. Reading participants’ transcripts, to get a sense of their general ideas in order to understand them fully.
2. Extracting “significant statements” from the narratives by identifying keywords and sentences relating to the phenomenon under study.
3. Formulating meanings for each of the significant statements extracted in (2) above.
4. Categorizing the participants’ experiences and recurrent statements into meaningful themes
5. Integrating the resulting themes into a rich description of the phenomenon under study. In this study, this step applied the constant comparative analysis method, which involves making systematic comparison across units of data (focus group discussions) to develop conceptualizations of the possible relations between various pieces of data (Boeije, 2002).
6. Reducing the themes to an essential structure that offers an explanation of the behaviour. To achieve this, narrative analysis, a method that recognizes the extent to which people provide insights about their lived experiences (Chase, 2005; Reisman, 2008), was applied.

The data analysis did not reveal any major variations in the experiences of young people by age and gender.

The overall study, from conceptualization to data analysis and dissemination, was undertaken in line with the core principles of ethical research involving human subjects. These principles include paying special attention to communicating the aims of the study, the rights of study participants such as the right to withdraw from the study at anytime, informed consent, confidentiality. To safeguard adherence to these principles, the study proposal, focus group guides, and consent forms were submitted to the Human Sciences Research Council of South Africa’s Research Ethics Committee (REC) review. The committee granted the study ethical clearance as per Protocol No. REC 13/19/02/14. The paper is being published in accordance with the conditions set out in the Data User’s agreement of the National Department of Health, the government entity that, with other organizations, commissioned the larger study.

## Results

Within the structural violence framework, a key finding from the FGDs was that healthcare workers often used their power and authority, morality and cultural accounts to reproduce a ubiquitous system of invisible obstacles that indirectly discouraged young people’s access to sexual and reproductive health services. Key among those related to social stigma were Healthcare workers perceived condescending attitudes and inappropriate questions; Healthcare workers’ delegitimising of AYP’s utilization of certain sexual and reproductive health services; the politics of age; perceived disregard of confidentiality. Finally, the analysis describes the emotional consequences of seeking sexual and reproductive health services in health facilities including away from AYP’s own communities.

### Healthcare workers’ condescending attitudes

Consistent with findings from previous South African studies (Jonas et al., 2017) healthcare workers’ perceived condescending attitudes were a recurring theme in discussions with AYP. The general view was that healthcare workers are unfriendly, unhelpful, and their manner of speaking to young people is seen as disrespectful. As the following excerpts suggest, such attitudes often discourage AYP from seeking sexual and reproductive services from public health facilities.

For me it is very difficult because of the attitude that we get from the [nursing] sisters; they don’t speak well with some of us and if I come here and I am not well-received or they don’t speak well with me, the next time I can’t come to the place where I know I will not be treated good (Male, 15–19 focus group)

It is not like I am blaming them, but they refuse to help with family planning; it is also just the manner in which they address us and how they speak with us (Female, 20–24 years focus group).

The nurses should learn to communicate well with people because we end up getting angry and not coming to the clinic or losing our respect for them, and when that happens it is like we have no respect for them, whereas they are the ones who are mistreating us (Female, 20–24 years focus group).

Another recurring contention was that young people did not often receive relevant information from providers. Instead, healthcare workers were fond of asking questions that young people perceived as embarrassing and inappropriate. The AYP variedly described such questions as “being lectured to”, “interrogated” and being “asked ridiculous questions”. For example:

Nurses are not friendly especially to us young people, we are sometimes asked questions that make us feel ashamed, like whether we had sex when we go for pregnancy testing and HIV testing. I was once asked that question and that made me feel upset (Female, 15–19 focus group).

When you come to get condoms, you are asked: “what you are going to do with them?” That question is embarrassing and it makes it difficult for shy people to go and get condoms (Male, 15–19 years focus group).

I went to the clinic … I wanted to test for pregnancy, they asked me why I’m testing for pregnancy; am I sleeping with guys? I said I’m not sleeping with guys and they told me to go home and why do I want to test for pregnancy if I’m not sleeping with guys. I felt very ashamed because of that (Female, 15–19 years focus group).

It is evident from the foregoing that such questions did not only make AYP feel disrespected and/or evoked negative feelings such as shame and embarrassment in them but the young people lamented that the questions somehow undermined their decision-making capacity, as the following statement illustrates:

I think the services are relevant for us but the problem is [that] before they can help you, you are being ‘lectured to’ about how young you are and the like, I think they should just help us and stop asking us why do we come to the clinic at young age (Female, 20–24 years focus group).

### The politics of age

While many young people, like the one in the statement above, felt that the sexual and reproductive health services they sought were relevant for them, many healthcare workers seem hold a different view which that AYP are too young to be accessing such services. The discussions with AYP revealed that these “politics of age” (Worthington et al., 2008) is another factor within the public health system that discourages young people from seeking sexual and reproductive health services, as the following statement illustrates:

Like we said, the clinic staff has an attitude towards us because some of the girls when they come for contraceptives the clinic staff ask them funny questions like ‘at your age are you having sex’. That makes it difficult for us to come to the clinic now. The clinic staff is not making it easy for us (Male, 20–24 years focus group).

This framing of young people as undeserving users of sexual and reproductive health services was also seen as healthcare workers’ underestimation of young people’s ability to assess their needs and adopt help-seeking behaviour. Indeed, some AYP stated that they sometimes disregard the healthcare workers’ advice to not utilize sexual and reproductive health services and/or dismissed it as interference by adults who doubted young people’s thinking abilities. For example:

I am old enough and I know what I am doing, whether or not I am using a condom is not her business. (Female, 20–24 years focus group).

For many AYP, however, questions about their age appropriateness made them feel judged and embarrassed and a result, many reported that they chose not to access sexual and reproductive health services.

### Healthcare workers’ disregard for confidentiality

Adolescent and young people reported that they visited the facilities primarily for services such as contraception, including condoms, HIV testing, pregnancy testing, treatment for sexually transmitted infections. With these services closely associated intimate sexual activities, young people would understandably prefer to access the services in strict confidence, and many expressed their desire to not have their parents or guardians know about their visits to the facilities for such services. To this end, the widespread perception that providers often disclose sexual and reproductive health-related information about young people outside the provider–client relationship emerged as one of the key barriers to AYP’s utilization of such services.

Now the problem is that the clinic staff takes confidential information and shares it with parents and that makes us not want to come to the clinic (Male, 20–24 years focus group).

This perceived breach of confidentiality was deemed to be particularly more prevalent in smaller communities where “everyone knows everyone”. For example:

The worst thing is when they know you or your relatives … They will be like ‘you are so young: does so and so know that you are here’. The result is that the next time you feel like coming to the clinic you feel that there is no use because sometimes you don’t want your parents to know that you are coming to the clinic. So, what’s the use if they end up knowing and asking you why you went to the clinic? (Female, 20–24 years focus group).

Overall, most AYP reported that they are often anxious that providers are not always discreet in handling “sensitive” information, and that this made them stay away from utilizing sexual and reproductive services. It also emerged that those who have the financial and time resources to travel sometimes sought the services from health facilities that were outside their communities or localities. For example:

As the youth of this village some of us do not use this clinic, we go to other villages because we don’t want people to know our problems (Male, 15–19 years focus group).

There is no privacy here, you can come here for testing [HIV] and the nurse will talk about you to the clinic staff, and the news spread, and everyone will know … It’s better to go to clinic where you are unknown (Female, 20–24 years focus group).

### Healthcare workers attitudes as structural violence

Within the framework of South Africa’s enabling legislative and policy framework for the provision of sexual and reproductive health services, AYP who seek such services explicitly and implicitly expect to be served by healthcare workers whose guiding ethic is to do no harm and whose interest will be to anchor young people as they transition from predominantly paediatric to health services for young adults. However, as the previous section has shown, in their attempts to access sexual and reproductive health services AYP often meet various forms of social stigma from healthcare workers. Taken together, the healthcare workers’ attitudes and behaviours that comprise this stigma demonstrates the existence of structural violence in the provision of sexual and reproductive health services for AYP in public health facilities. As earlier alluded to in the paper, structural violence is not crime-related form of violence; it is ethics-related and can take the form of psychological, physical and social harm. This, when healthcare workers use their power and authority to deny young people access to sexual and reproductive health services, the emotional discomfort or distress that is created and experienced as, for example, shame, embarrassment and/or feeling disrespected constrains AYP’s agency to be responsible for their safe sexuality.

Healthcare workers perceived discriminatory attitudes and practices towards AYP seeking sexual and reproductive health services based on their age is also a violation of the young people’s right to access these services, to receive reliable information on the services, and to live without fear and anxiety knowing that their sexual and reproductive needs can be met with dignity. Young people whose health needs in this regard are not met either because they could not tolerate being “interrogated” about their sexual behaviour and as a result chose not to seek the services are essentially being exposed to the risk of negative sexual and reproductive health outcomes such as sexually transmitted infections including HIV, unplanned pregnancies, unsafe abortions, etc.

development and notion suggesting, for example, that this age cohort is too young to use sexual and reproductive health services is not only misinformation, but it also has the potential to socially harm young people because it bolsters the social norms against utilization of sexual and reproductive health services at a young age. This is particularly the case because the mechanisms through which healthcare workers overlook the uniqueness of AYP’s situation and miss opportunities for engaging and counselling these young people in defining and meeting their health needs often creates social stigma towards them. The utterances that AYP associated with their experiences in this regard depicted contexts that did not encourage demand for these services through reassurance, information and provision of requited services. Rather, AYP assert, healthcare workers often delegitimised young people sexual and reproductive health needs. It was shared, for example, that were instances where some healthcare workers complained in a hostile manner that AYP take most of the workers time as they requested for certain services. For example:

I was there for family planning and that nurse; I didn’t like her manners. I didn’t like the way she was addressing me … I came for HIV test and she told me that all the girls she had been testing were for pregnancy and HIV the whole day. She said she was doing the same task for the whole day and that she was tired of testing pregnant girls and she was going on about why we were not using condoms.

These contestations for knowledge and power between healthcare workers and AYP about which sexual and reproductive health services were legitimate and deserving of healthcare workers’ time, and who has first-hand knowledge about the validity AYP’s needs undermine collaboration between the two parties and potentially place AYP in harm’s way. In essence, it can undermine primary or early intervention and is associated with experiences of distress in healthcare settings.

Some AYP who perceived confidentiality to be lacking in their community sexual and reproductive health facilities sought services away from their local communities or localities. neighbourhoods. To the extent that such strategies entailed incurring time and monetary costs on travel their uptake can lead to delays in seeking help and/or treatment and support and AYP’s experience of structural violence as they spend time wondering how to navigate the resource obstacles.

Overall, the perceptions about providers’ hostility and negative attitudes create a provider–patient relationship that is not conducive to providing AYP with requisite sexual and reproductive health services and the context of these interactions leads to healthcare workers missing opportunities that constitute harm placed on AYP’s way, and have far-reaching consequences for their health and overall wellbeing. Even if these actions may not be intended, it can be argued that they present a certain degree of deprivation of health. The emotional hurt and embarrassment experienced by AYPs in such circumstances bring into the focus the realization that “there is no reason to assume that structural violence amounts to less suffering than personal violence” (Galtung, 1969, p. 173) and it results in the preventable diminishing of vital human needs or … the impairment of human life itself, which reduces the ability of someone to meet their needs below that which would otherwise be imaginable (Farmer et al., 2006; Galtung, 1969).

## Discussion and conclusion

This paper explored South African AYP’s experiences of seeking sexual and reproductive health services in public health facilities in the country. The findings largely mirror those of previous that have illuminated an array of discriminatory and hostile attitudes from public healthcare providers towards young people seeking sexual and reproductive health services (Chandra-Mouli et al., 2015; Kleinert & Horton, 2016; **authors**). Much of this previous research has attributed these attitudes to, *inter alia*, service providers’ poor skills of dealing with AYP (Svanemyr et al., 2015) and the stigma often associated with young people’s use of sexual and reproductive health services (Jewkes et al., 2009). To this end, a new contribution by this paper is that healthcare workers in public faculties often use their power to place a ubiquitous system of invisible obstacles that discourage AYP from using sexual and reproductive health services.

These obstacles, and the mechanisms through which they are presented, neatly fit the description of structural violence, given by Galtung (as cited in Farmer et al., 2006, p. 1686), as the “avoidable impairment of fundamental human needs or … the impairment of human life, which lowers the actual degree to which someone is able to meet their needs below that which would otherwise be possible”. It can thus be argued that when healthcare workers do not adequately listen to needs of young people visiting healthcare facilities for sexual and reproductive health services and, instead, downplay these needs to the extent that the young people are discouraged or too irritated to continue seeking services, crucial opportunities to attain positive sexual and reproductive health outcomes through early intervention can be scuppered. Such behaviour by

Healthcare workers can be described as unethical and in violation AYP’s right to access basic health services in dignity It also places the young people in the way of harm way by exposing them to the risk negative health outcomes such as early and unwanted pregnancy and sexually transmitted infections.

Given the sensitivity of the adolescence phase of life and centrality of the self-concept in social relationships, questioning AYP’s wisdom in seeking sexual and reproductive health services can be a threat to their healthy sexual development. Indeed, the global recognition that investments in adolescence and young people should be at the centre of development is based on the understanding that positively influencing young people during this phase has broad consequences for minimizing risks to their health and wellbeing, their future adult life and even for future generations (Kleinert & Horton, 2016). Conversely, when they are not supported with reliable information and education and when their health seeking actions are counteracted by healthcare workers, AYP often fail to exercise their agency and their rights to access available sexual and reproductive health services and, in consequence, contribute to the burden of poor sexual and reproductive health outcomes to population and development (WHO, 2014; Jaca et al., 2018; Sridhar et al., 2014).

In South Africa class, racism, and sexism are common aspects of discrimination that are often considered when analysing public health issues (Rylko-Bauer & Farmer, 2016). This study revealed that in addition to these common aspects, AYP in the country also face an implicit form of discrimination—such as age, morality and cultural accounts—that some healthcare workers use under the disguise of moral guidance to discourage sexual activity at young ages (Jewkes et al., 2009). Denying young people access to sexual and reproductive health services due to these latter aspects not only undermines the legislative and policy frameworks that guide South Africa in the promotion of AYP’s health and development, but they are also a form of structural violence as they place the health of young people in harm’s way (Farmer et al., 2006).

All in all, and consistent with previous studies (Chandra-Mouli et al., 2015; Kleinert & Horton, 2016), this paper demonstrated that hostility was a common encounter between service providers and young people seeking sexual and reproductive health services in South African public health facilities. The overall conclusion of the paper is that when healthcare workers use their authority and power to influence AYP’s trajectories of accessing sexual and reproductive health services in negative ways they violate AYP’s right to health now and in the future. As Galtung points out, in the contexts of structural violence, the harm to people does not have to be direct and interpersonal; “The violence is built into the structure and shows up as unequal power and consequently as unequal life chances” (Galtung, 1969, p. 171). This overall situation contravenes South Africa’s legislative and policy framework directing that young people should be provided with timely, free, and requisite sexual and reproductive health facilities (Beksinska et al., 2014).

Our understanding of human rights also informs us that the experiences of AYP in relation to healthcare workers’ attitudes entail, to a large extent, oppression and dehumanization of young people who depend on the public health sector in South Africa. Although these experiences have been widely researched under the rubric of “barriers to access to sexual and reproductive health services” (Abubakari et al., 2020; Ninsiima et al., 2021), this understanding has not led to much radical changes in these services. A concept such as structural violence helps us see the abnormalities inherent in healthcare practices that persist in the context of human rights framed policies and the “efficacy of the concept of structural violence lies in its ability to render visible the social machinery of oppression” (Farmer, 2004, p. 319).

This paper’s findings, therefore, underscore and reaffirm the recommendations of previous studies (e.g., Jewkes et al., 2009; Schriver et al., 2014; Geary et al., 2014, 2015; **authors**) calling for attitudinal training and sensitization among sexual and reproductive health providers in South Africa. Such training should be aimed at, among other matters, breaking down prejudices including based on age, cultural norms about AYP’s sexual behaviour, ensuring that young people are recognized as sexual beings, and ensuring that all service provision is made in strict alignment with the prevailing national and international legislative and policy guidelines to create an enabling socio-cultural environment that supports the public investments made to realize positive sexual and reproductive health outcomes in South Africa.

To the extent that structural violence is not about physical manifestations of suffering or impairment only, but about social arrangements including institutions that “put individuals and populations in harm’s way and it can almost be invisible because it is widespread and ‘normalised by stable institutions and regular experience” (Farmer et al., 2006, p. 1686). Future research should explore direct and indirect sexual and reproductive health outcomes given that structural violence produces social inequalities through social control and oppression of the less powerful, in this case AYP. This includes exploring the various mechanisms used by healthcare providers to delegitimize utilization of sexual and reproductive health services by AYP and how to enhance reporting and accountability at all levels with the view to support mainstreaming of provision of sexual and reproductive health services in routine healthcare of AYP.
